# Force enhancement in lengthening contractions of cat soleus muscle in situ: transient and steady-state aspects

**DOI:** 10.1002/phy2.17

**Published:** 2013-06-28

**Authors:** Ryan A Koppes, Walter Herzog, David T Corr

**Affiliations:** 1 Department of Biomedical Engineering, Rensselaer Polytechnic InstituteTroy, New York; 2 Human Performance Laboratory, University of CalgaryCalgary, Alberta, Canada

**Keywords:** Force enhancement, history dependence, skeletal muscle, stretch activation

## Abstract

Force enhancement (FE) associated with lengthening is a well-accepted phenomenon of active skeletal muscle, but the underlying mechanism(s) remain unknown. Similar to force depression (FD) following active shortening, the mechanism of FE may be attributed, at least in part, to cross-bridge kinetics. To examine this relationship, a post hoc analysis was performed on the transient force relaxation phase of previous in-situ FE experiments in soleus muscle-tendon units of anesthetized cats. For each muscle (*n* = 8), nine eccentric lengthenings (3 amplitudes, 3 velocities) were performed while tetanically stimulated (3T at 30 Hz, 3× α motorneuron, 35 ± 1°C). To determine transient aspects of FE, the period immediately following stretching was fit with an exponential decay function (*R*^2^ > 0.95). Statistical analyses revealed that total steady-state FE (FE_SS_) increased with stretching amplitude and applied mechanical work. A positive relationship was observed between the active FE_SS_ and rate of force decay *(k),* indicating that a kinetic mechanism may explain active FE. However, for all muscles and stretch conditions, there was no correlation between the total amount of FE_SS_ and rate of decay. Therefore, FE cannot be explained solely by an active FE mechanism involving the interaction of actin and myosin. Rather, these findings suggest a combination of underlying mechanisms, including a kinetic mechanism for active FE, contributions of a passive elastic element, and possibly an activatable passive component operating outside of actin–myosin cross-bridging. Moreover, this transient analysis identifies that FE is not simply the opposite of FD, and its underlying mechanism(s) cannot simply be the opposite in nature.

## Introduction

Steady-state force enhancement (FE_SS_) is defined as an increase in steady-state force following an activated lengthening compared to that of an isometric contraction at the corresponding final length (Abbott and Aubert 1952; Edman et al. 1982; Rassier 2012). This enhancement of isometric force after lengthening is a well-accepted characteristic of active skeletal muscle demonstrated in whole muscle (Ruiter et al. 2000; Lee and Herzog 2002), single-fiber preparations (Edman et al. 1978, 1982; Sugi and Tsuchiya 1988), single myofibrils (Ranatunga et al. 2007; Joumaa et al. 2008a; Joumaa and Herzog 2010), and single sarcomeres (Leonard et al. 2010). Force enhancement (FE) has been observed in skeletal muscle of insects, amphibians (Julian and Morgan 1979; Edman and Tsuchiya 1996), and mammals (Hisey et al. 2009; Leonard et al. 2010), as well as voluntary contractions in humans (Hahn et al. 2007, 2010; Oskouei and Herzog 2009; Shim and Garner 2012). Despite being well characterized experimentally, the underlying mechanism(s) of this phenomenon remain unknown.

Previous work has amply demonstrated the correlation between increasing FE_SS_ and increasing amplitudes of stretch (Abbott and Aubert 1952; Edman et al. 1978, 1982; Sugi and Tsuchiya 1988; Cook and McDonagh 1995) on both the descending limb (Edman et al. 1978, 1982; Morgan et al. 2000) and the ascending limb (Herzog and Leonard 2002; Hisey et al. 2009; Rassier and Pun 2010) of the force-length relationship. Unlike force depression (FD) in which the amount of steady-state FD (FD_SS_) increases with decreasing shortening speeds, no clear relationship between FE_SS_ and the velocity of stretch has been observed (Edman et al. 1982; Sugi and Tsuchiya 1988; Lee and Herzog 2008b).

The possible underlying mechanism(s) for FE continue to be highly debated (Herzog et al. 2006; Morgan and Proske 2006; Rassier 2012). Morgan and colleagues proposed the sarcomere nonuniformity theory to explain the increase in force as dispersion of sarcomere lengths in response to the stretch on the descending limb of the force-length relationship of muscle (Morgan et al. 2000). In this theory, when subjected to active lengthening, some of the weaker sarcomeres will “pop,” elongating down the descending limb until their passive force equals the active force of the stronger sarcomeres in series. To accommodate the overall length change, the stronger sarcomeres will not need to lengthen as much because the weak sarcomeres stretch considerably. This dispersion in sarcomere length between the highly elongated weaker sarcomeres and the shorter, stronger sarcomeres results in a higher force than if the sarcomeres lengthened uniformly. This is an elegant model, capable of explaining the observations of both FD and FE for increasing amplitudes of shortening or stretch, respectively.

Herzog and colleagues have challenged the sarcomere nonuniformity theory by demonstrating the presence of FE on the ascending limb (Peterson et al. 2004; Pun et al. 2010; Rassier and Pun 2010) and within single sarcomeres (Joumaa et al. 2008a; Leonard et al. 2010; Pun et al. 2010). Furthermore, no clear example of sarcomere popping has ever been observed (Pun et al. 2010; Rassier 2012; Rassier and Pavlov 2012), and the distribution of sarcomere lengths after active lengthening has been monitored in isolated myofibrils (Telley et al. 2006a,b) and a single sarcomere (Pavlov et al. 2009). For all these investigations of FE by the Rassier group and Telley et al., the nonuniformity of sarcomere lengths were tracked and could not be identified as the sole mechanism responsible for FE following eccentric stretch. In light of this, much attention has been focused on the proposed mechanism in which the increase in force production is the result of a parallel elastic element (Edman et al. 1978; Herzog and Leonard 2002; Pinniger et al. 2006). Acting as a calcium-dependent molecular spring, titin has been suggested to function in parallel to the cross-bridges to produce FE_SS_ in lengthened active muscles (Horowits 1992; Granzier et al. 2000; Herzog and Leonard 2002). Although titin's contribution has been documented, it cannot account for the entirety of the observed higher force (Bullimore et al. 2008; Joumaa et al. 2008a).

To date, the current understanding of FE is primarily limited to steady-state observations. Previous investigations of ramp velocities and amplitudes on cross-bridge kinetics have been restricted to the period of activated lengthening and peak force traces (Bagni et al. 2005; Pinniger et al. 2006; Colombini et al. 2007; Roots et al. 2007), but few investigations have addressed what occurs following lengthening in active muscle (Kosterina et al. 2012). The work of Rassier and Herzog has demonstrated a link between cross-bridge kinetics and FE (Rassier and Herzog 2005). Further analysis of transient phenomena may provide novel insight into the possible kinetic mechanisms associated with FE. More specifically, by examining the transient force recovery phase, the role of cross-bridge kinetics and their possible influence on FE might be revealed. Our previous work investigating the transient aspects of FD following active shortening has shown that the rate of force redevelopment correlated with FD_SS_, suggesting a strong kinetic component to the FD mechanism. A strong argument has been made to link FD_SS_, cross-bridge kinetics (Herzog 1998; Corr and Herzog 2005), and stress-induced inhibition of cross-bridge binding (Marechal and Plaghki 1979; Huxley et al. 1994). As with the sarcomere nonuniformity theory, a similar mechanism, but opposite in nature, might therefore be partially responsible for FE.

To identify the transient aspects of FE and their relation to stretching speed, stretching amplitude, and mechanical work done on the muscle, we performed a post hoc analysis of previous in situ experiments in soleus muscle tendon units of anesthetized cats (Herzog and Leonard 2002). The period immediately following lengthening, during which the force returns to steady-state, was fit using an exponential decay function. The aims of this study were to analyze and quantify the effects of stretching amplitude, stretching speed, and muscle mechanical work on both FE_SS_ and the transient force relaxation rate after active lengthening. The results of this study were interpreted with respect to prior FD experiments (Corr and Herzog 2005), to identify if the two phenomena exhibited similar transient and steady-state behaviors, and thus could be described by similar underlying mechanism(s).

## Materials and Methods

### Experimental

**Figure 1:**
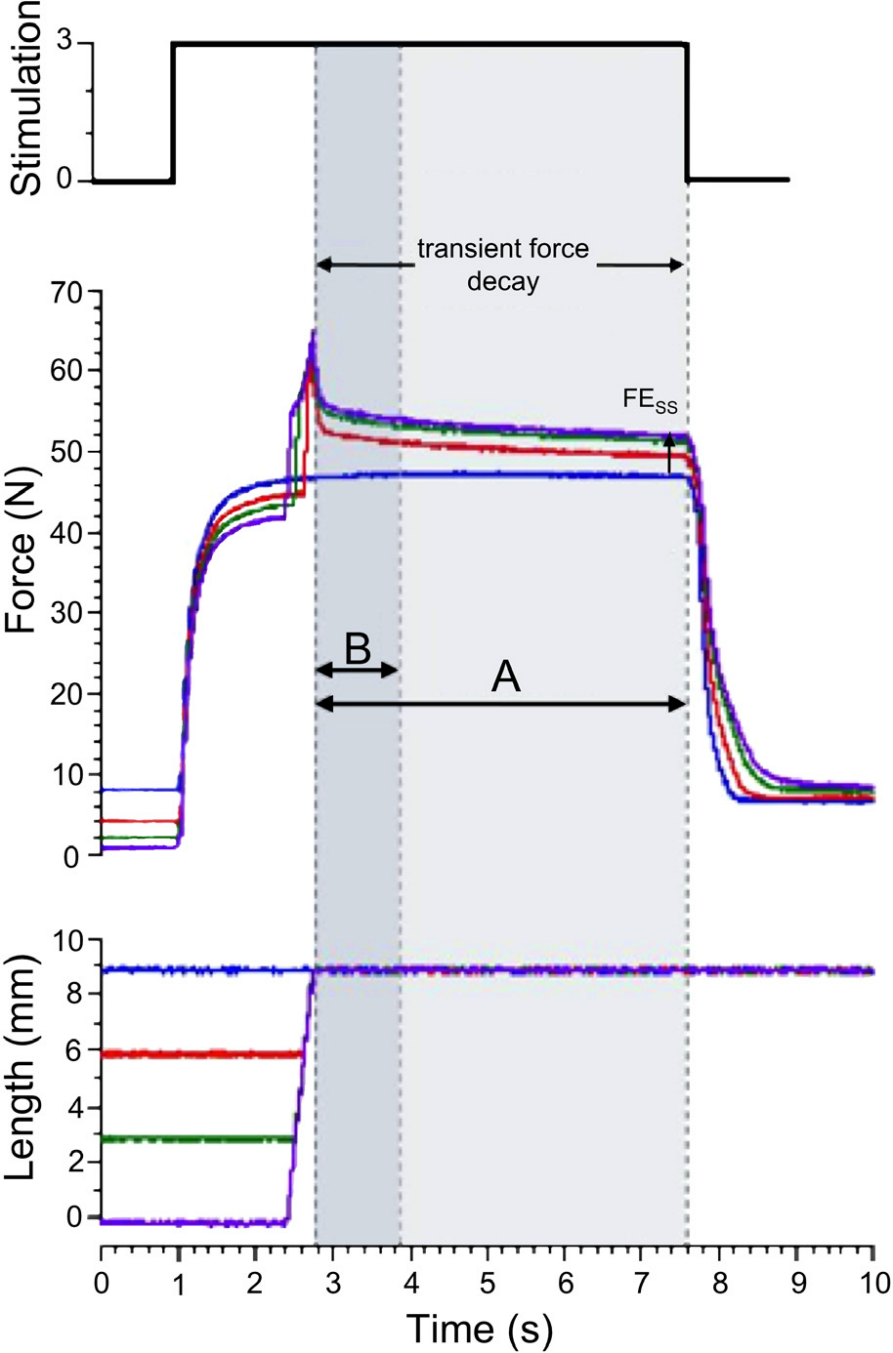
Representative force-time data with corresponding stimulation-time (top) and length-time (bottom) traces for a subset of the test battery: three different amplitudes of stretch (3, 6, 9 mm), at one constant stretching speed (27 mm/sec). Each force-time trace is composed of tetanic stimulation (3T, 3× α motoneuron threshold) at initial length, an active lengthening phase, and a period of transient force decay. For this post hoc analysis, only the period of transient force decay following stretch was analyzed. The entire transient recovery phase (A: 0–4.9 sec following stretch) was fit to obtain values of *F*_inf_ (*R*^2^ > 0.91). The early transient recovery period (B: 0–1.0 sec following stretch) was fit (*R*^2^ > 0.95) to determine the amount of force decay, *A*, and the decay rate, *k*. Steady-state force enhancement (FE_SS_ = *F*_inf_ − *F*_iso_), the difference between the force of the test contraction at steady state and the corresponding isometric reference contraction, is shown symbolically for the 9-mm stretch.

A post hoc analysis was conducted on previously described FE experiments (Herzog and Leonard 2002) on the descending limb of in situ cat soleus muscles (*N* = 8). In these experiments (Herzog and Leonard 2002), FE following active lengthening was found by using methods approved by the Life Sciences Animal Ethics Committee of the University of Calgary. Stretch amplitude (3, 6, 9 mm; corresponding to approximately 3–9% of the total muscle length and approximately 7–21% of optimal fiber length) and stretch velocity (3, 9, 27 mm/sec; i.e., approximately 7, 21, and 63% of optimal fiber length/sec) were systematically varied (Fig. 1). All tests were performed at a stimulation frequency of 30 Hz. The entire 4.9-second isometric period following active lengthening was analyzed, in which the muscle was held at the final length while tetanically stimulated (3T, 3× alpha motorneuron threshold). Muscle temperature was maintained at 35 ± 1°C.

### Transient analysis

To quantify the transient force decay, *F(t)*, the force-time data after activated lengthening were analyzed using an exponential relaxation function,


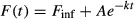
(1)

where *A* is the amount of force decay (difference between steady-state force (*F*_inf_) and the initial force immediately after stretching), and *k* is the exponential force relaxation rate. Values of *F*_inf_, *A*, and *k* were obtained for each experiment by fitting the entire 0–4.9 sec of force-time data after stretching with equation (1) (*R*^2^ > 0.91) using a Levenberg-Marquardt error minimization algorithm in a commercial curve-fitting program (DeltaGraph 4.0, SPSS Inc., Chicago, IL). Fits obtained in this period were biased toward the steady-state force, which resulted in accurate estimates of *F*_inf_, but underestimated values of *k*, as evidenced by poor agreement in the early transient response. To obtain more accurate early transient parameter estimates, the model was fit to the first 1.0 sec of force-time data after stretch, yielding larger *k* estimates, lower *F*_inf_ estimates, similar estimates of *A*, and improved transient agreement (*R*^2^ > 0.95). Across all lengthening protocols and muscles, fits to both the initial 1.0 sec and the entire 4.9 sec yielded similar amounts of force relaxation (*A*), indicating that nearly all force decay occurred within the first 1.0 sec following stretch. Therefore, values of *k* and *A* were obtained from the early transient fits (0–1.0 sec) and *F*_inf_ values from the entire transient recovery period (0–4.9 sec). Total FE_SS_ was found as the difference between *F*_inf_ and steady-state isometric force (FE_SS_ = *F*_inf_ − *F*_iso_). This is similar to the methods previously employed by Corr and Herzog (2005) to study transient force redevelopment following active shortening. The passive component of FE_SS_ was found as the difference between actively lengthened traces and the isometric trace at the same final length, each evaluated 3.0 sec after deactivation (Herzog and Leonard 2002). Subsequently, the passive component was subtracted from the total FE_SS_ to find the active contribution to FE_SS_ (FE_SS_ = active FE_SS_ + passive FE_SS_). The mechanical work performed to actively lengthen the muscle was determined by integrating the force-displacement trace.

### Statistical analysis

Statistical analyses were conducted to observe the relation between the controlled variables and both steady-state and transient aspects of the force after activated lengthening. A block design ANOVA was employed to determine the significance between the effects of stretch speed and stretch amplitude on transient (*k*, *A*) and steady-state variables (total and active FE_SS_). Regression analyses, performed across individual muscles as well as for all muscles pooled together, were used to explore the correlations between measured quantities (FE_SS_, *A*, *k*) and the amount of mechanical work, as well as between transient (*k*) and steady-state (total and active FE_SS_) aspects of FE.

## Results

**Figure 2:**
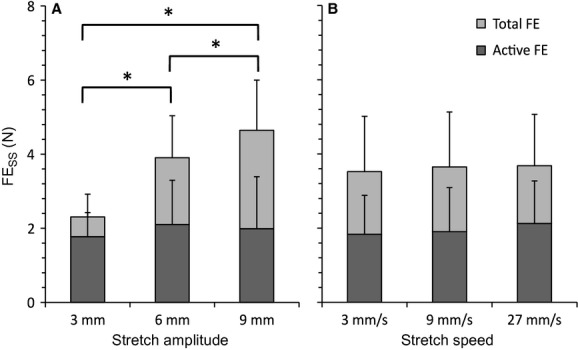
The total (light gray bars) and active FE_SS_ (dark gray bars) with respect to stretching amplitude (pooled for all speeds, *n* = 24 per amplitude; A), and stretching speed (pooled for all amplitudes, *n* = 24 per speed; B), indicating a significant increase in the total FE_SS_ with increasing amplitudes of stretch, and no significant correlation between total FE_SS_ and increasing speeds of stretching. The average active FE_SS_ remained relatively constant, showing no change with either the amplitude or speed of shortening. Data are estimated marginal means ± SD of the means, with significance determined by complete block ANOVA, *P < 0.05.

**Figure 3:**
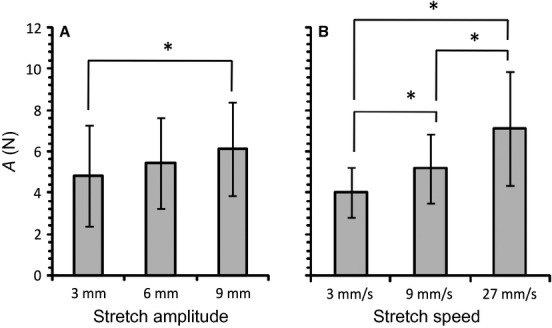
The amount of decayed force (*A*) with respect to stretching amplitude (pooled for all speeds, *n* = 24 per amplitude; A), and stretching speed (pooled for all amplitudes, *n* = 24 per speed; B), indicating a significant change in force from the smallest to largest amplitude of stretch, and a significant decayed force increase with increasing speeds of stretching. Data are estimated marginal means ± SD of the means, with significance determined by complete block ANOVA, *P < 0.05.

**Figure 4:**
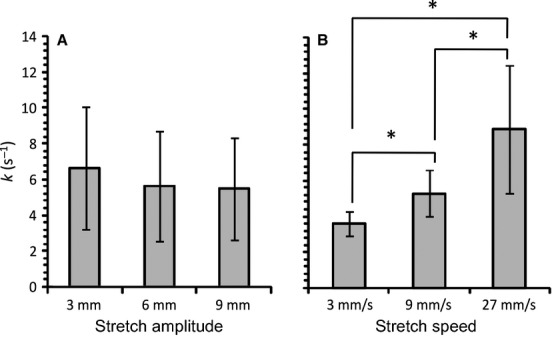
The force decay rate (*k*) with respect to stretching amplitude (pooled for all speeds, *n* = 24 per amplitude; A), and stretching speed (pooled for all amplitudes, *n* = 24 per speed; B), indicating no significant correlation to changing amplitudes of stretch, and a significant increase in decay rate with increasing speeds of stretching. Data are estimated marginal means ± SD of the means, with significance determined by complete block ANOVA, *P < 0.05.

**Figure 5:**
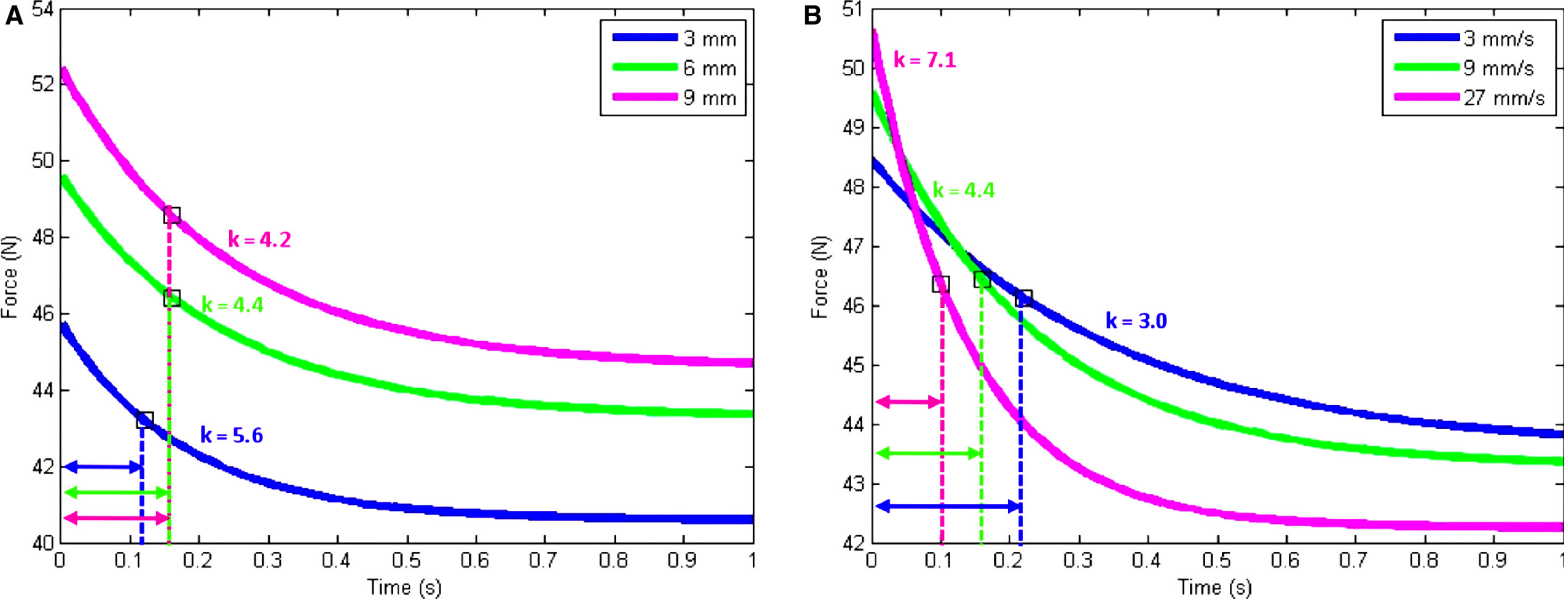
Representative plots of an individual muscle, illustrating the effect of stretching amplitude (A; speed = 9 mm/sec) and stretching speed (B; amplitude = 6 mm) on the force decay rate, *k*. In both panels, the vertical dashed lines indicate the time required to decay to 50% of the force (i.e., *A*/2), illustrating a weak correlation between recovery rate and increasing amplitudes of stretch (A), and a strong increase in the decay rate with increasing stretching speed (B).

As demonstrated previously, the total amount of FE_SS_ increased with increasing stretching amplitudes (Fig. 2A), but had no correlation to stretch speeds (Fig. 2B). The active component of FE_SS_ showed no significant difference with either speed or amplitude of stretch, and remained fairly constant across all test conditions (Fig. 2). Thus, the observed increase in FE_SS_ with stretch amplitude is dominated by the passive component of FE_SS_ (Fig. 2A). The amount of force decay, *A*, increased slightly with the amplitude of stretch (Fig. 3A), and showed a clear increase with increasing speeds of stretch (Fig. 3B). The rate of force decay, *k*, showed no clear correlation with increasing stretch amplitudes (Fig. 4A), but significantly increased with increasing speeds of stretch (Fig. 4B). This was also observed within each single muscle; the force decay rate (*k*) was not influenced by the amplitude of stretch (Fig. 5A), but was strongly influenced by the speed of stretch (Fig. 5B). Despite the relative insensitivity of *k* to changing amplitudes of stretch, the muscles (both individually and when pooled together) tended to exhibit slower force decay when subjected to longer stretch amplitudes, although this difference in rate was relatively small and not statistically significant.

**Figure 6:**
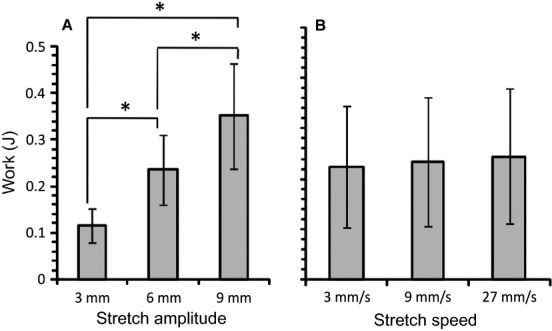
Mechanical work imposed on the muscle during stretching with respect to stretching amplitude (pooled for all speeds, *n* = 24 per amplitude; A), and stretching speed (pooled for all amplitudes, *n* = 24 per speed; B), indicating a significant increase in work with increasing amplitudes of stretch, and no significant correlation between work and increasing speeds of stretching. Data are estimated marginal means ± SD of the means, with significance determined by complete block ANOVA, *P < 0.05.

**Figure 7:**
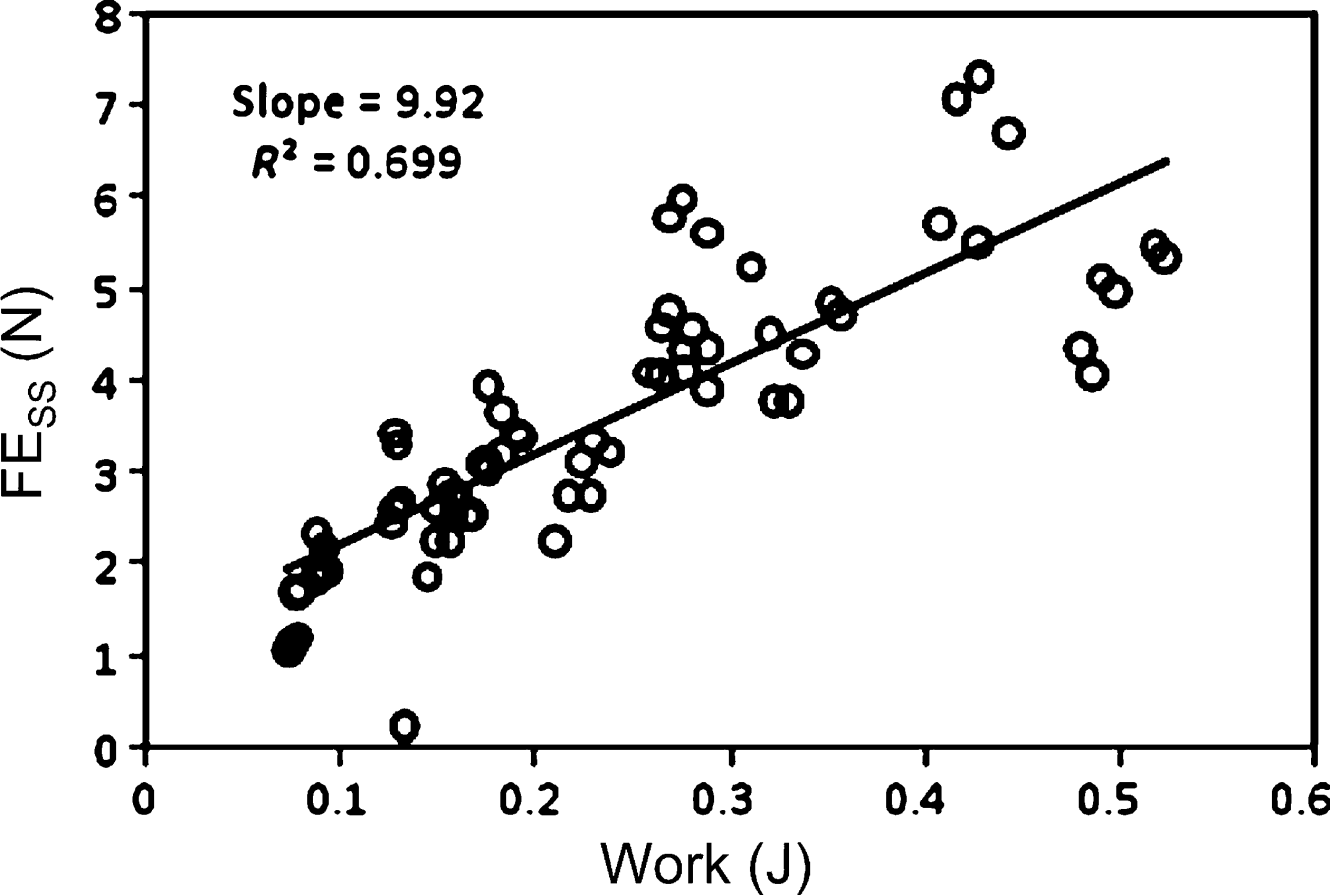
Regression analysis demonstrates a significant positive relationship between total FE_SS_ and the mechanical work imposed on the muscle during stretching (*P* = 0.014).

**Figure 8:**
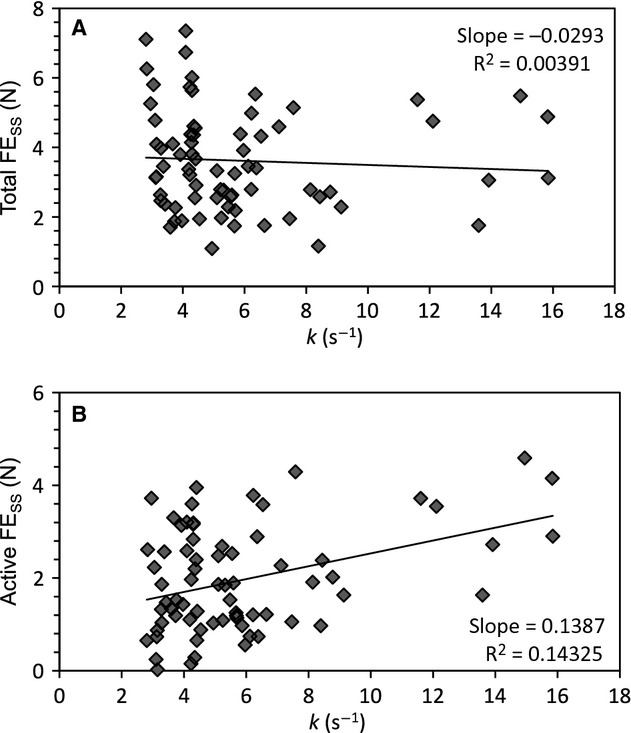
Regression analysis between the rate of force decay (*k*) and both the total steady-state force enhancement (FE_SS_); A), and the active component of FE_SS_; B). No clear correlation exists between the transient decay rate and the total FE_SS_ (*P* = 2.25), but when the passive component of FE_SS_ is removed, *k* possesses a positive relationship with active FE_SS_ (*P* < 0.01).

Mechanical work increased significantly with increasing amplitudes of stretch (Fig. 6A), and demonstrated no clear trend with increasing speeds of stretch (Fig. 6B). When pooled for all muscles and conditions, mechanical work significantly correlated positively with FE_SS_ (*R*^2^ = 0.699, Fig. 7, *R*^2^ = 0.857 summed for Individual muscles, not shown) and *A* (*R*^2^ = 0.377, *R*^2^ = 0.731 summed for individuals muscles, not shown). However, neither mechanical work nor FE_SS_ significantly correlated to the rate of force decay, *k* (not shown and Fig. 8A, respectively). When the passive component was subtracted from the total FE_SS_, yielding the active component, a positive correlation between the rate of decay and active FE_SS_ was observed (Fig. 8B).

## Discussion

While the underlying mechanism(s) of FE remain unknown, titin and other passive elements within the sarcomere have been investigated for their potential contribution to the mechanism of FE. Bullimore et al. (2008) showed that a parallel elastic element could not be solely responsible for the higher force seen in the presence of stretching in activated single fibers of the frog. Furthermore, Joumaa et al. (2008b) demonstrated in a contractile-less myofibril that titin could only account for approximately 25% of the residual passive FE. Thus, a passive mechanism for FE cannot account for all of the increase in force observed after active lengthening. For this reason, an underlying active mechanism, possibly involving the cross-bridging interaction of myosin and actin, may also contribute to FE (Rassier and Herzog 2005; Herzog et al. 2012). Rassier and Herzog (2004) showed that the addition of a cross-bridge inhibitor (2,3-butanedione monoxime, BDM) increased total FE while decreasing the isometric force, thereby suggesting a kinetic mechanism for FE. In a follow-up study (Lee and Herzog 2008a), a similar increase in FE was observed with decreasing temperatures. As, in both situations (addition of BDM and lower temperatures) cross-bridges are biased toward the weakly bound state, the authors suggested FE to be related to an increase in weakly bound cross-bridges. Roots et al. (2012) further demonstrated that the amount of FE_SS_ decreases with increasing temperatures, and their cross-bridge modeling suggested FE_SS_ to be the combination of strain of noncross-bridge components, and a reduced cycling rate due to strain on slowly cycling cross-bridges. Joumaa and Herzog (2013) further demonstrated a decrease in ATPase activity following active lengthening, implying that activated skeletal muscle becomes more efficient following stretch, and substantiating a possible role of actin in the mechanism of FE. As previously shown in Corr and Herzog's (2005) investigation of FD, clues of an active mechanism may lie in the transient force recovery period. Our transient analysis identified a positive correlation between the active component of FE_SS_ and the rate of decay, such that greater active FE_SS_ was accompanied by a faster relaxation of force. We, however, found no such link between the total amount of FE_SS_ and the rate of force decay. Thus, FE is likely a combination of kinetic and passive contributions; an active FE that is influenced by actin–myosin interaction, and a more dominant, passive FE mechanism that operates outside of cross-bridge kinetics.

We analyzed the transient force relaxation period following active lengthening to investigate a correlation between the rate of decay and both the total amount and active component of FE. With excellent agreement (*R*^2^ > 0.95), the exponential decay function (eq. 1) provided function parameters representative of important quantities of the force decay: the amount of decayed force (*A*), the enhanced force at steady-state (FE_inf_), and the exponential rate of decay (*k*).

**Figure 9:**
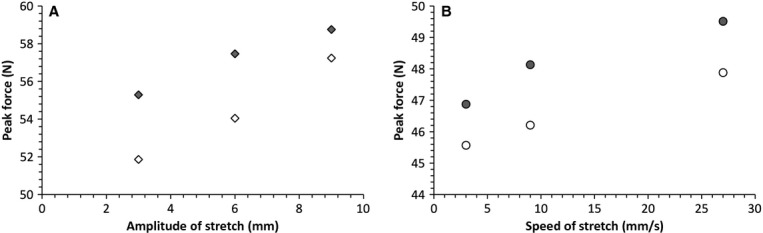
The peak force measured immediately after active lengthening for the collateral muscles of a representative animal, showing greater peak forces with increasing amplitudes and speeds of stretch. (A) For increasing amplitudes of stretch at a constant speed of 9 mm/s the peak force following active stretch increases for both the left (⋄) and right (♦) soleus within an individual cat. (B) The peak force following stretch increases for increasing velocities of stretch of a constant amplitude of 6 mm for both the left (○) and right (•) soleus of an individual animal, indicating that more force is applied to the muscle. This suggests the selected velocities lie on the dynamic portion of the eccentric force-velocity relationship. Results are typical of all animals tested.

The amount of decayed force (*A*) significantly increased with increases in both stretch amplitude and stretching speed (Fig. 3). This increase in *A* implies an increase in the force at the end of stretch for larger magnitudes of stretch, regardless of stretching speed. In all muscles tested, the peak force after active lengthening was greater for increasing amplitudes of stretch (Fig. 9A) and faster speeds of stretch (Fig. 9B). Recent work by Edman (2012) makes the argument that the force during stretch is independent of stretch amplitude. However, Bullimore et al. (2007) demonstrated a clear correlation between the force at the end of stretch and the amount of FE_SS_ for increasing magnitudes of stretch. Our findings show that longer stretches (to the same final length) result in an increase in force at the end of stretch, and an increase in recovered force (Figs. 1, 3A), thereby suggesting that FE is built up during active lengthening, possibly by a passive elastic element. This is supported by our observation that nearly all of the increase in FE_SS_ with increasing amplitudes of stretch is attributable to the passive component (Fig. 2A). Taken together with our transient analyses, this suggests that in addition to an active component of FE that correlates with an increasing decay rate, there exists a passive element contribution that is engaged upon activation, providing a mechanism of FE that operates outside of the actin–myosin cross-bridge kinetics.

Like that of previous work, a positive relationship between the total FE_SS_ and stretch amplitude was demonstrated (Abbott and Aubert 1952; Edman et al. 1978; Sugi and Tsuchiya 1988; Cook and McDonagh 1995), and no significant correlation was shown between FE_SS_ and stretch speed (Edman et al. 1978, 1982; Lee and Herzog 2008b), thereby demonstrating the consistency between the results of this work and previous work on FEss. Furthermore, as previously shown, we observe a passive FE that correlates with stretch amplitude following deactivation of the muscle (Herzog and Leonard 2002; Herzog et al. 2003; Lee and Herzog 2008b). A strong correlation exists between the mechanical work done on the muscle and the total FE_SS_ (Fig. 7) (Kosterina et al. 2009). However, across all muscles and perturbations, no correlation exists between the rate of force decay and the total FEss (Fig. 8A).

We observed, as in previous studies, that the amount of FE_SS_ was not significantly influenced by the speed of stretch. However, if the muscle velocities chosen for this investigation fell upon the plateau of the eccentric force-velocity curve, the force would remain constant for all stretch speeds explored, thereby masking a potential influence of force (and work) during stretch. If the underlying mechanism of FE were dependent on the mechanical work applied to the muscle, and the force during lengthening remained constant for all experimental velocities, no correlation would be expected between FE_SS_ and the speed of stretch. Our results demonstrate a significant increase in applied mechanical work (Fig. 6) for increasing speeds of stretching (3, 9, and 27 mm/sec) across all magnitudes of stretch, and for each individual muscle, the peak force following stretch increased for faster stretches (Fig. 9B). Furthermore, to assure the selected muscle velocities did not fall on the plateau of the force-velocity curve, the speeds of stretching were compared to previously reported force-velocity data collected on the cat soleus muscle. Sandercock and Heckman's (1997) force-velocity data, in addition to our results showing greater peak force with increasing stretching speed (Fig. 9B), clearly demonstrates that the selected velocities of stretching fall on the dynamic phase of the force-velocity curve. Therefore, the force applied to the muscle during lengthening changes with increasing speeds of stretch, and validates our results indicating that total FE_SS_ does not correlate with the speed of stretch nor with the rate of recovery, *k*. The lack of correlation between total FE_SS_ and *k* supports the presence of a mechanism(s) outside the interaction of myosin and actin, such as a parallel elastic element, the nonuniformity of sarcomere lengths (Rassier 2012) or a mechanism involving an interaction of actin and titin (Herzog et al. 2012). In addition to titin, other sarcomeric proteins such as nebulin (Chandra et al. 2009), myosin-binding protein C (Knoll 2012), and desmin could play a role in FE.

Our previous FD studies in cat soleus showed that the rate of force redevelopment following active shortening negatively correlated with the magnitude of FD_SS_ and mechanical work, such that an increase in mechanical work resulted in a slower rise to a more depressed steady-state force, thereby suggesting a kinetic mechanism of FD. The results of this investigation found that active FE_SS_ correlates to the relaxation rate, suggesting that active FE has, at least in part, a kinetic component. However, active FE was relatively constant throughout these experiments, indicating the insensitivity of this active FE to the amplitude of length changes. Moreover, no clear correlation was found between the rate of force relaxation or applied mechanical work and the total amount of FEss. Thus, while a kinetic component may be present in the FE mechanism, its contributions to total FE appear relatively small compared with passive, noncross-bridge contributions. Therefore, unlike FD, this study supports the suggestion that FE is, in large part, the result of a passive mechanism outside of the cross-bridge. As a result, these findings suggest that FE cannot simply be explained by a similar mechanism to that of FD, but opposite in sense. To gain a more fundamental understanding of the mechanism(s) of this history-dependent behavior, future investigations of FE may require accurate methods for probing the proteomics of the sarcomere in functional muscles to systematically test the influence of both active and passive components.
